# No differences in physical activity between children with overweight and children of normal-weight

**DOI:** 10.1186/s12887-020-02327-y

**Published:** 2020-09-09

**Authors:** Janneke van Leeuwen, Bart W. Koes, Winifred D. Paulis, Patrick J. E. Bindels, Marienke van Middelkoop

**Affiliations:** 1 Department of General Practice, Erasmus MC, University Medical Center, Wytemaweg 80, P.O. Box 2040, 3000 CA Rotterdam, The Netherlands; 2 Department of Physical Therapy Studies, Rotterdam University of Applied Sciences, Rotterdam, The Netherlands

**Keywords:** Physical activity, Overweight, Children

## Abstract

**Background:**

The aim of this study was to investigate the differences in objectively measured physical activity and in self-reported physical activity between overweight and normal-weight children.

**Methods:**

Data from a prospective cohort study including children, presenting at the participating general practices in the south-west of the Netherlands, were used. Children (aged 4–15 years) were categorized as normal-weight or overweight using age- and sex specific cut-off points. They wore an ActiGraph accelerometer for one week to register physical activity, and filled out a diary for one week about physical activity.

**Results:**

A total of 57 children were included in this study. Overweight children spent significantly less percentage time per day in sedentary behavior (β − 1.68 (95%CI -3.129, − 0.07)). There were no significant differences in percentage time per day spent in moderate to vigorous physical activity (β 0.33 (− 0.11, 0.78)). No significant differences were found between children of normal-weight and overweight in self-reported measures of physical activity.

**Conclusions:**

Overweight children are not less physically active than normal-weight children, which may be associated with the risen awareness towards overweight/obesity and with implemented interventions for children with overweight/obesity.

## Background

Childhood obesity is one of the most serious public health challenges of the twenty-first century, according to the World Health Organization [1]. It can, among other diseases, lead to pulmonary complaints, diabetes, and cardiovascular diseases like hypertension [2]. Besides reducing sedentary time, and promoting a healthy diet, increasing physical activity is another strategy to address childhood obesity. Therefore, to fight the childhood obesity epidemic, and promote other health benefits, children are advised to be moderately to vigorously physically active for at least 60 min each day [1].

Previous literature states that children with overweight and obesity are less physically active than children of normal-weight based on objective data of accelerometers [3, 4]. However, these studies are conducted over 10 years ago, while since then several initiatives have been launched to reduce overweight and obesity. In the Netherlands (in 2010), the ‘covenant healthy weight’, promoting healthy lifestyle for children, was introduced [5]. The covenant healthy weight aimed to increase awareness on the health risks of overweight and obesity, and consequently decrease the prevalence of overweight and obesity in the Netherlands. Therefore, the research question investigated in this manuscript is whether children with overweight and obesity in today’s society are as active, or, as hypothesized, less active than their normal-weight peers.

Physical activity and sedentary behavior can be measured objectively with accelerometers or inclinometers, but the usage of diaries and questionnaires is also often used. This way of data collection is subjective and the validity of self-reported physical activity by children and parents is controversial [6]. Moreover, it has been shown that parents of children with overweight overestimate their child’s physical activity [7]. Though, self-reported questionnaires are a valid methodological approach to measure sedentary behavior in adolescents [8].

The aim of this study is to describe potential differences between children with overweight and children of normal-weight in objectively measured and self-reported physical (in)activity.

## Methods

### Study design

This study is a longitudinal cohort study with a follow-up of one week using a subsample from the DOERAK (Determinants of (sustained) Overweight and complaints; Epidemiological Research among Adolescents and Kids in general practice) study [9]. The DOERAK study is a prospective cohort study including 733 children with a two-year follow-up, that studied differences between children with and without overweight that consulted the general practitioner (GP) [10]. The DOERAK study was primarily designed to study differences between with- and without overweight, in the number of consultations at the GP, the type of complaints, quality of life, and levels of physical activity. The study has been approved by the Institutional Review Board of the Erasmus University Medical Center, Erasmus MC (MEC-2010-092).

### Participant selection for DOERAK database

Children aged 2–18 years, visiting one of the 71 participating GP practices located in various socio-economic regions in the South-West of the Netherlands between December 2010 and April 2013 with any type of complaint were invited to participate in the study. They, or their parents, had to have at least a basic understanding of the Dutch language. Children with mental or physical disabilities, children with serious co-morbidities affecting weight and children consulting their GP with emergency problems were not invited to participate in the study. If children showed interest to participate in the study and after verbal consent, height, weight and waist circumference of the child were measured by the GP, and contact information of the parents was gathered. For assessing height and weight, calibrated height and weight measures were used by the GP who received instructions and followed an identical protocol [9]. Waist circumference was measured midway between the lowest rib and the top of the iliac crest, at the end of gentle expiration. Parents then received written study information and an informed consent form (children aged 12 years and older also received an informed assent form). The child was formally included in the study when the signed informed consent (and signed informed assent form when needed) was received by the research team.

### Subsample selection for current study

From the 733 included children in the DOERAK cohort, it was aimed to ask every fifth child with overweight and fifteenth child of normal-weight aged 4–18 years to wear an accelerometer for one week (ActiGraph, GT3X, Pensacola, Florida) to provide objective information about sedentary time and physical activity [11]. These 65 children were used for the current study. The cut-off of a minimum age of 4 years old was used, since this is the age that children in the Netherlands start attending elementary school, and can join sport clubs.

### Data collection

After formal inclusion, the researcher sent a questionnaire to the GP to collect data on the child’s age and sex, and the GP measured height, weight and waist circumference during consultation. Parents of included children received a questionnaire to collect data on demographics (i.e. socio-economic status, highest education in household, ethnicity, marital status) of parents and child. Children aged 9 years and older received an online diary which had to be filled out once each day in the same week the ActiGraph was worn. For younger children (aged 4–9 years), parents were asked to fill out the diary. The diary contained open questions on how many hours were spent on sleeping, watching tv, playing sports, outdoor play, and using the computer. There was also room for comments about taking off the ActiGraph during sports/showering. Children had to wear the ActiGraph at the waist at the right side of the body for seven days; five weekdays and two weekend days. Epoch length was set at 10 s. The measurement started at 7 am and ended 8 days later at 7 am. Children were instructed to take off the ActiGraph when going to bed and with activities involving water. The first full seven days of recording were used for the analysis. The child received the activity monitor the day before the measurements started in order to become familiar with the device. The researcher or research assistant who delivered the ActiGraph to the participants gave instructions to children and their parents on how to wear the ActiGraph. Children and parents were asked to put on the accelerometer as soon as the child woke up.

### Measures

The GP questionnaire was used to extract child’s age and sex and from height and weight measurements, BMI-z scores were calculated and weight status was determined using the international age and sex specific cut-off points [12]. This international standard was used, since it is based on international data and linked to the widely accepted adult cut off point for overweight and obesity of 25 and 30 kg/m^2^ respectively [12]. This makes it easy to compare the current data set to other international datasets. Since only a small percentage of the included children was obese (*n* = 3), children with overweight (*n* = 19) and obesity were combined into one category called the ‘overweight’ category. Parent’s questionnaires were used to extract information on baseline demographics. Socio-economic status (SES) was based on net household income, and was dichotomized into ‘low SES’(< 2000 Euros/month) and ‘middle/high SES’(≥2000 Euros/month). Ethnicity (‘both parents born in the Netherlands’ and ‘at least one parent not born in the Netherlands’), and marital status (‘parents are together’ and ‘parents separated’) were also dichotomized. Parental education was categorized into three levels: ‘up to lower level secondary education’, ‘higher level secondary education’ and ‘at least a bachelor diploma’.

The diary was used to extract data on the amount of hours per day spent on watching TV, using the computer, outdoor play and playing sports. The outcome measures were categorized into: 0 = not applicable, 1 = 30 min or less, 2 = 30 min - 1 h, 3 = 1–2 h, 4 = 2–4 h, 5 > 4 h.

Data from the ActiGraphs were extracted using ActiLife (v.5.4.0.0). Non-wearing time was defined as a period of at least 20 min of consecutive zero counts [13]. ActiGraph data were considered valid when daily wearing time was at least 8 h a day and if there were at least 3 valid weekdays and 1 valid weekend day. Children who wore the ActiGraph less than this predetermined amount of days were excluded from analyses (*n* = 6). For children with valid ActiGraph data, all valid weekdays and weekend days were used in the analyses. The chosen cut-off points in counts per minute (cpm) for the various activity levels were < 100 cpm for sedentary behavior, < 2220 cpm for light, < 4136 cpm for moderate and ≥ 4136 cpm for vigorous activity [14]. The amount of time spent in each level of activity per day, and the percentage of time spent in each activity level per day (time spent per day in level of activity/total wear time of that day) were extracted from ActiLife (v5.4.0.0).

The percentage of time spent per day in moderate and vigorous activity were clustered into ‘moderate to vigorous physical activity’ (MVPA).

### Sample size calculation

Based on the formula of Fleiss [34] with a two-sided significance level of 0.05 and a power of 90% and the median result of 580 cpm in a day from Riddoch et al. [37], 50 participants in both the normal-weight- and the overweight group are needed to find a difference of 10% between the groups [9, 15, 16].

### Statistical analysis

Descriptive statistics were used to describe baseline demographics. Potential differences between children of normal-weight and with overweight were tested using independent t-tests.

In order to account for differences between children in total wear time of the ActiGraph per day, percentages of time spent in the different activity levels were used as outcome measures. Percentage of time spent in each level of physical activity was a continuous variable. Potential differences in sedentary behavior and physical activity between children with overweight and children of normal-weight were tested using linear mixed models. Effects of mixed model analyses were expressed as the percentages of time spent in activity level per day of children with overweight compared to children of normal-weight; expressed as beta (β), with accompanying 95% confidence intervals (CI). Generalized estimating equations (GEE) were used to test for differences between children with overweight and of normal-weight for self-reported time spent on watching TV, using the computer, playtime outside and playing sports. To examine differences between percentage of children with overweight and of normal-weight meeting the WHO guidelines of 60 min of MVPA per day, GEE was used. Effects were expressed in β, with 95%CI. All analyses were adjusted for sex, age and ethnicity. The significance level was set at *p* < 0.05.

## Results

### General characteristics

Of the 65 children with ActiGraph data, six were excluded from the analyses due to invalid wear time. Weight status was missing for two children because of missing height and/or weight at baseline. Therefore, 57 children were included in the current study, of which the characteristics are presented in Table 1. The average age of the participating children was 8.7 (3.2) years and 60% was female. Of the participating children, 24% had a family with a low socio-economic status and in 19% of the children, at least one parent was born in another country than the Netherlands. In 19% of the families, the parents were separated and in 15% of the families, the highest level of education from the parents was up to lower secondary level. In families of the children with overweight, compared to children of normal-weight, significantly more often one parent was born in another country.

**Table 1:** Participant characteristics

	Study population ***N*** = 57	Normal-weight ***N*** = 35	Overweight ***N*** = 22	***p***-value
Age, mean (SD), y	8.7 (2.5)	8.4 (2.7)	9.1 (2.2)	0.4
Sex: female, N (%)	34 (59.6)	20 (57.1)	14 (63.6)	0.3
Socio-economic status	**N(%)**	**N(%)**	**N(%)**	
Low (< 2000)	12 (23.5)	8 (25.0)	4 (21.1)	0.004
Middle/High (> = 2000^)	39 (76.5)	24 (75)	15 (78.9)	< 0.001
Highest education in household	**N(%)**	**N(%)**	**N(%)**	
Low (up to lower secondary level)	8 (15.1)	5 (15.6)	3 (14.3)	0.08
Middle (upper secondary level)	21 (39.6)	10 (31.3)	11 (52.4)	< 0.001
High (at least bachelor level)	24 (45.3)	17 (53.1)	7 (33.3)	0.005
Ethnicity	**N(%)**	**N(%)**	**N(%)**	
Both parents born in Netherlands	43 (81.1)	28 (87.5)	15 (71.4)	< 0.001
At least one parent born in another country	10 (18.9)	4 (12.5)	6 (28.6)	0.005
Marital status	**N(%)**	**N(%)**	**N(%)**	
Parents separated	11 (19.3)	8 (25.0)	3 (14.3)	0.08
Parents together	42 (73.7)	24 (75.0)	18 (85.7)	< 0.001

### Actigraph-data

**Fig. 1:**
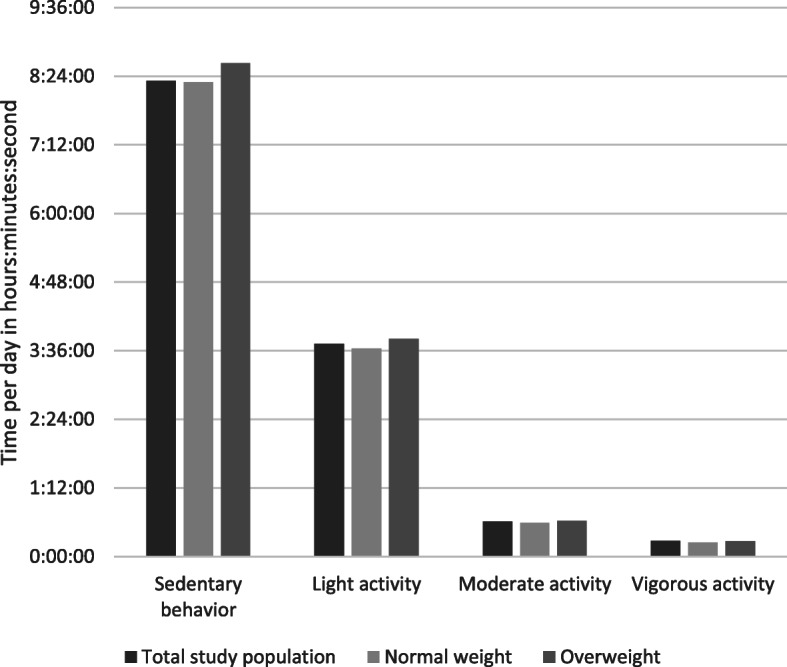
Time per day spent in each level of physical activity for the total study population, normal-weight children and children of overweight

On average, children had 4.7 valid weekdays and 1.9 valid weekend days on which they wore the ActiGraph for at least 8 h. The average total wear time per day was 12 h, 51 min and 4 s (sd 02:48:24). Figure 1 shows the amount of time per day spent in each level of physical activity for the total study population, and for normal-weight and children with overweight separately. Children with overweight spent significantly less percentage time per day in sedentary behavior (β − 1.68 (95%CI -3.29, − 0.07)). There were no significant differences between children of normal-weight and overweight in percentage time per day spent in light activity (β 1.26 (− 0.06, 2.59)), and in MVPA (β 0.33 (− 0.11, 0.78)) (Table 2).

**Table 2:** Results of the linear mixed model analyses on the influence of weight status on % of time spent per day in each level of activity

	Mean % (sd)	Beta	95% C.I.	P-value
% time in sedentary				
Normal-weight	50.6 (6.7)	Ref		
Overweight	57.8 (5.9)	−1.68	−3.29 - -0.07	0.04*
% time in light activity				
Normal-weight	2.2 (5.4)	Ref		
Overweight	6.8 (4.8)	1.26	−0.06 – 2.59	0.06
% time in moderate to vigorous activity				
Normal-weight	0.5 (2.4)	Ref		
Overweight	0.5 (2.2)	0.33	−0.11 – 0.78	0.14

All analyses were adjusted for sex, age and ethnicity. *p < 0.05

The number of children meeting the WHO guidelines of at least 60 min of MVPA per day based on objective measured data ranged per day from 24.1–39.3% (data not shown). On average per week, 27% of children of normal-weight and 37% of children with overweight met the WHO guidelines on physical activity, based on objectively measured data (data not shown). No significant difference was seen between the number of children of normal-weight and with overweight meeting the WHO guidelines (β − 0.56 (− 1.18, 0.07)) [17, 18].

### Self-reported physical activity

No differences were seen between children of normal-weight and children with overweight for self-reported time spent on watching TV, using the computer, playtime outside and playing sports which was gathered from the diaries (Table 3).

**Table 3:** The average time per week spent on different types of physical activity

	Study Population (N = 57)	Normal-weight (N = 35)	Overweight (N = 22)
Time spent watching TV			
Not Applicable	14.3%	17.2%	10.3%
1/2 h or less	22.2%	18.3%	26.5%
½ - 1 h	26.0%	28.5%	23.9%
1–2 h	26.7%	24.2%	30.8%
2–4 h	9.5%	10.2%	7.7%
> 4 h	1.3%	1.6%	0.9%
Time spent on the computer			
Not Applicable	44.5%	45.0%	46.6%
1/2 h or less	24.5%	24.1%	19.8%
½ - 1 h	17.2%	16.8%	19.0%
1–2 h	11.3%	11.0%	12.9%
2–4 h	1.9%	2.1%	1.7%
> 4 h	0.6%	1.0%	0%
Time spent on playing outside			
Not Applicable	9.0%	9.3%	8.5%
1/2 h or less	11.6%	8.3%	17.1%
½ - 1 h	16.8%	18.7%	13.7%
1–2 h	23.2%	26.4%	17.9%
2–4 h	23.5%	24.4%	22.2%
> 4 h	15.8%	13.0%	20.5%
Time spent on playing sports			
Not Applicable	60.9%	62.1%	58.8%
1/2 h or less	5.9%	5.3%	7.0%
½ - 1 h	12.8%	11.6%	14.9%
1–2 h	15.8%	15.3%	16.7%
2–4 h	3.0%	5.3%	1.8%
> 4 h	0.5%	0.5%	0.9%

## Discussion

Children with overweight spent less percentage time per day in sedentary behavior (− 1,68%)compared to children of normal-weight. The magnitude of this difference is small, which is characterized by the following calculation: if a child of normal-weight would spent 6 h (=360 min) in sedentary behavior per day, a child with overweight would spend on average − 1.68% * 360 = 6.05 min less in sedentary behavior per day. Even though this difference is small, it indicates that children with overweight are certainly not less physically active, than children of normal-weight. Furthermore, no differences were seen in percentage time per day spent in light activity and MVPA between children of normal-weight and overweight. Self-reported data on physical activity, which was measured with a diary, also showed no differences in physical activity between children of normal-weight and overweight. On average, 73.3% of children of normal-weight and 63.2% of children with overweight, did not meet the WHO guidelines on daily physical activity based on objectively measured data [17].

In contrast with the current study, others found that children with overweight are less physically active than children of normal-weight, based on objective measurements [3, 4]. Also, children of lower ses, who are also more prone to be overweight, are found to be less physically active than children of higher ses [19, 20]. The finding of the present study, i.e. children with overweight are not less physically active than children of normal-weight may be associated with the risen awareness and implemented interventions for children with overweight and obesity. In the Netherlands, the ‘covenant healthy weight’ has resulted, among other things, in special health programs at schools and after-school physical activity intervention programs [5]. These implementations and the improved awareness may have resulted in higher activity rates in children with overweight. Though, it remains unclear whether these intervention programs affected the physical activity rates of the children included in this study.

The importance of meeting the WHO physical activity guidelines is highlighted by the benefits children gather from being physically active such as improved bone health, improved cognition, improved weight status, and reduced risk of depression [17, 21]. National data from the Netherlands, based on online- and paper questionnaires, found that in 2015, 48% of Dutch children, aged 4–12 years met the WHO guideline on physical activity [17, 22]. This number is slightly higher than the number found in the current study (24.1–39.3%) which used objective measures (rather than questionnaires) to measure physical activity. It could be suggested that questionnaires are less accurate than objectively measured data, due to, among other things, recall bias. A study by Verloigne et al. conducted in 2010 also used accelerometers to measure levels of physical activity in 10–12 year old children [23]. They found that 2.1% of the girls and 15.8% of the boys in the Netherlands met the WHO guidelines on physical activity [17, 23]. These numbers indicate that even though many interventions promoting physical activity in children (with overweight) are present these days, the number of children meeting the physical activity guideline are not sufficient yet.

There may also be other explanations for the fact that the children with overweight of the current cohort are not less physically active than children of normal-weight. The children with overweight in this cohort may be more focused on their weight and perhaps already motivated to change their lifestyle, since they were willing to participate in a study focused on overweight and obesity. The included children with overweight may have started to increase their level of physical activity as soon as the study started. Additionally, wearing an ActiGraph for a week is an intervention in itself, which could have resulted in higher physically active children with overweight compared to their normal-weight peers. These confounders mentioned may have an influence on the results, but cannot be controlled for.

### Strength and limitations

By assigning every fifth child with overweight and every fifteenth child of normal-weight to the subsample used in the current study, we tried to minimize selection bias. The subsample did not differ from the DOERAK cohort in basic demographics [10]. However, when we compare our subsample to the most recent numbers of the overall Dutch population, parents from our cohort were more highly educated (45.3% vs 32%) [24]. Furthermore, it could be suggested that the parents and children participating in the DOERAK study are more motivated to lose weight compared to the overall Dutch population, since the DOERAK study is a study about overweight and obesity. Therefore, our cohort might not be completely representative for all Dutch children, and it could have led to an overestimation of physical activity levels in the Dutch population.

The size of our study sample was smaller than intended [9]. The smaller sample size may have introduced a power problem. We found a difference in time spent on physical activity, but a larger sample size, introducing more variation in the demographics, could have had an impact on the effect we found in the current study. Therefore we believe that our results should be handled with care and further research with larger sample sized populations should be performed.

## Conclusions

In our study, children with overweight are not less physically active than children of normal-weight. However, the majority of both children of normal-weight and with overweight do not meet the guidelines of 60 min of MVPA per day. Therefore, promoting physical activity in all children should remain an important topic for today’s society.

## Data Availability

The datasets used and/or analysed during the current study are available from the corresponding author on reasonable request.

## Abbreviations

GP: General practitioner
cpm: counts per minute
MVPA: Moderate to vigorous physical activity
CI: Confidence interval
GEE: Generalized estimating equations
